# RNA m^6^A Methylation Regulators Subclassify Luminal Subtype in Breast Cancer

**DOI:** 10.3389/fonc.2020.611191

**Published:** 2021-01-29

**Authors:** Lin Yang, Shuangling Wu, Chunhui Ma, Shuhui Song, Feng Jin, Yamei Niu, Wei-Min Tong

**Affiliations:** ^1^ Department of Pathology, Institute of Basic Medical Sciences, Chinese Academy of Medical Sciences, Beijing, China; ^2^ School of Basic Medicine, Peking Union Medical College, Beijing, China; ^3^ Molecular Pathology Research Center, Chinese Academy of Medical Sciences, Beijing, China; ^4^ Department of Breast Surgery, The First Affiliated Hospital of China Medical University, Shenyang, China; ^5^ China National Center for Bioinformation, Beijing, China; ^6^ National Genomics Data Center & CAS Key Laboratory of Genome Sciences and Information, Beijing Institute of Genomics, Chinese Academy of Sciences, Beijing, China

**Keywords:** RNA methylation, m^6^A regulators, genomic regulation, breast cancer subtypes, subclassification, survival

## Abstract

RNA N^6^-methyladenosine (m^6^A) methylation is the most prevalent epitranscriptomic modification in mammals, with a complex and fine-tuning regulatory system. Recent studies have illuminated the potential of m^6^A regulators in clinical applications including diagnosis, therapeutics, and prognosis. Based on six datasets of breast cancer in The Cancer Genome Atlas (TCGA) database and two additional proteomic datasets, we provide a comprehensive view of all the known m^6^A regulators in their gene expression, copy number variations (CNVs), DNA methylation status, and protein levels in breast tumors and their association with prognosis. Among four breast cancer subtypes, basal-like subtype exhibits distinct expression and genomic alteration in m^6^A regulators from other subtypes. Accordingly, four representative regulators (IGF2BP2, IGF2BP3, YTHDC2, and RBM15) are identified as basal-like subtype-featured genes. Notably, luminal A/B samples are subclassified into two clusters based on the methylation status of those four genes. In line with its similarity to basal-like subtype, cluster1 shows upregulation in immune-related genes and cell adhesion molecules, as well as an increased number of tumor-infiltrating lymphocytes. Besides, cluster1 has worse disease-free and progression-free survival, especially among patients diagnosed with stage II and luminal B subtype. Together, this study highlights the potential functions of m^6^A regulators in the occurrence and malignancy progression of breast cancer. Given the heterogeneity within luminal subtype and high risk of recurrence and metastasis in a portion of patients, the prognostic stratification of luminal A/B subtypes utilizing basal-featured m^6^A regulators may help to improve the accuracy of diagnosis and therapeutics of breast cancer.

## Introduction

Breast cancer is the most ubiquitous cancer in women worldwide. It is a heterogeneous disease and has been classified into different subtypes according to the gene expression profile. These subtypes are termed as human epidermal growth factor 2 (HER2)-enriched, basal-like, and luminal subtypes (1, 2). Standardization of breast cancer classification and optimal treatment regimens for each subtype have acquired great progress since the concept of subtype was first proposed. Patients of HER2-enriched subtype benefit from HER2-targeted therapy, such as trastuzumab and pertuzumab (3). In contrast, patients of basal-like subtype have poor prognosis, high risk of recurrence, and lack efficient therapeutic strategy (1, 4, 5). Luminal subtype, accounting for 70% of breast cancers, has positive response to endocrine therapies and the best prognosis (6). However, substantial heterogeneity still exists within this subtype (7–9). By utilizing immunohistochemical analysis of progesterone receptor (PR) and Ki-67, luminal subtype could be further classified into less-aggressive luminal A and more-aggressive luminal B subtypes (10). Despite that, the standards of PR status and Ki-67 index remain controversial across the world or even among hospitals. Additionally, there also exists undeniable intragroup heterogeneity bringing about indeterminacy in clinical management (7, 11). Thus, continuous efforts have been made to subclassify the intrinsic subtypes into more precise subgroups. For instance, basal-like cancers were further classified into 6 (12) or 4 (13) subgroups based on their genomic and transcriptomic profiling. By taking advantage of DNA copy number, DNA methylation, and gene expression data, luminal subtype was successfully segregated into subgroups with distinct molecular and clinical characteristics (8, 9, 11, 14). Nevertheless, despite the extensive investigations on breast cancer, genetic variance still brings about different responses to standard treatment protocol within the same subtype. Therefore, it is important to comprehensively understand the regulatory mechanism of gene alterations in pathological status.

Other than extensively studied genomic, transcriptomic, and epigenetic modulations, RNA m^6^A modification (m^6^A) represents a vital layer of epitranscriptomic regulation of gene expression and has drawn much attention in recent years. m^6^A is the most prevalent epitranscriptomic modification in mammals. Its formation is catalyzed by methyltransferase complex (also called “writers”), which is composed of core components METTL3, METTL14 (15–17), WTAP (18), and other subunits. Conversely, RNA m^6^A methylation can be removed by demethylases, better known as “erasers,” specifically FTO (19) and ALKBH5 (20). The effects of m^6^A on gene expression are mediated by m^6^A binding proteins which are usually called “readers.” So far, m^6^A regulators have been unveiled to function in regulating RNA alternative splicing, nuclear export, degradation, and translation (21). Given their crucial roles in many different physiological contexts, aberrant expression of m^6^A regulators could incur the occurrence or progression of multiple cancers through disturbing the m^6^A-dependent RNA metabolism (22).

Aberrant expression of m^6^A regulators, including METTL3, METTL14, WTAP, ALKBH5, and FTO, has been identified in breast cancer, as well as their potential prognostic values (23, 24). Mechanistically, upregulation of METTL3, METTL14, and FTO expression exhibits oncogenic roles by promoting cells proliferation, migration, or invasion in m^6^A-dependent manner (25–28). On the other side, hypoxia-dependent expression of ALKBH5 and ZNF217 is associated with the maintenance and specification of breast cancer stem cells *via* their inhibitory role on m^6^A methylation of mRNAs encoding pluripotency factors NANOG or KLF4 (29, 30). Despite the progress in the above regulators, there is still a lack of comprehensive analysis to excavate the roles and clinical applications of all the known m^6^A regulators in breast cancer.

Recently, bioinformatics analyses provide convenient tools in identifying the m^6^A regulators applicable in tumor classification and prognosis prediction in multiple cancers (12, 31–35). In this study, we analyzed the molecular alterations of m^6^A regulators and found their distinctive features in breast cancer. Besides, survival analysis revealed the prognostic values of several m^6^A regulators in breast cancer. Our results suggest their critical roles in the initiation and progression of breast cancer and diverse regulatory mechanisms of them. Furthermore, according to the DNA methylation status of 11 probes located on basal-like subtype-featured m^6^A regulators, luminal A and luminal B subtypes were further segregated into two clusters respectively, which differed in the enrichment of tumor infiltrating lymphocytes (TILs) and patients’ prognosis. Subclassification of luminal subtype will provide additional prognostic information in an attempt to improve personalized treatment of breast cancer.

## Materials and Methods

### Data Collection

Six types of breast cancer datasets (**Table S1**) originated from the Cancer Genome Atlas (TCGA) database (36) were downloaded from UCSC xena platform (37): the gene expression profiles obtained were originally generated from the Illunima HiSeq 2000 platform and transformed into log_2_(RSEM+1) format; somatic mutation data was compiled in Mutation Annotation Format (MAF); gene-level copy number variations (CNVs) were measured experimentally using the Affymetrix Genome-Wide Human SNP Array 6.0 platform and preprocessed with GISTIC2 method (38); DNA methylation levels estimated by beta values were measured based on the GPL13534 platform (Illumina Infinium HumanMehytlation450 Bead-Chip array). The beta values of DNA methylation are continuous variables between 0 and 1, representing the percentage of methylated alleles; miRNA expression data was generated from IlluminaHiseq platform, while the miRNA-target interactions were downloaded from miRTarBase database (39); phenotype data contained the survival and subtype information of each sample.

Proteomic datasets were obtained from another two independent studies. The first proteomic study applied a quantitative liquid chromatography/mass spectrometry-based proteome analysis to 65 breast tumors and 53 adjacent non-cancerous tissues (40). This dataset was used for comparing expression between tumor and normal samples. The other study utilized high-resolution accurate-mass tandem mass spectrometry method and contained 105 breast tumors with explicit information of subtyping and prognosis, which was applied for comparison among subtypes and for survival analysis (41).

### Correlation Analysis

The Pearson correlation coefficients between gene expression and DNA methylation, copy number, or miRNA expression were computed in R with *cor.test* function, respectively. Only the DNA methylation probes that had missing values in less than 50% of samples were included for the analysis. Different versions of miRNA IDs were converted through *miRBaseVersions.db* R package (42).

### Determination of Basal-Featured m^6^A Regulators

The importance of m^6^A regulators in distinguishing basal-like samples from other subtypes was ranked by performing random forest algorithm based on their gene expression levels. This procedure was processed in R with *RandomForest* package (43). Furthermore, the variable selection was determined by using *varSelRF* R package (44).

### Samples Clustering Analysis

Based on phenotype data, only normal samples and breast tumor samples allocated to explicit subtypes were included in sample clustering analysis. t-Distributed Stochastic Neighbor Embedding (t-SNE) analysis was performed with the expression values of all 28 m^6^A regulators using the *tsne* R package (45). Unsupervised hierarchical clustering analysis was performed with filtered DNA methylation probes, whose beta values should meet the below criteria (1): absolute value of Pearson correlation coefficient with gene expression was greater than 0.3; (2) standard deviation (SD) among all samples was higher than 0.2. Consensus clustering that determined the number of clusters for luminal and basal-like samples was implemented with *ConsensusClusterPlus* package in R by resampling iteration (50 iterations, resampling rate of 80%). The cluster number was determined according to the relative change in area under the cumulative distribution function (CDF) curve (46). The heatmap corresponding to the consensus clustering was generated with *pheatmap* R package.

### Differential Expression Analysis

Differentially expressed genes between cluster1 and cluster2 samples were defined with *DESeq2* R package (47). Briefly, the original log_2_(RSEM+1) values were transformed into RSEM values and grounded to integers, then the expression matrix was imported using the *DESeqDataSetFromMatrix* function. Genes that met the criteria of adjusted *P* < 0.05 and FoldChange > 1.5 or < 0.66 were regarded as differentially expressed genes between cluster1 and cluster2 samples.

### Functional Enrichment Analysis

Kyoto Encyclopedia of Genes and Genomes (KEGG) pathway enrichment analysis of the differentially expressed genes between cluster1 and cluster2 samples was implemented with *clusterProfiler* R package (48). Marker genes for each immune cell population were curated from published research (49), the relative abundance of different types of TILs in each sample was assessed by single sample gene set enrichment analysis (ssGSEA) method (50) in *GSVA* R package.

### Statistical Analysis

All analyses were implemented with R computing framework (v3.6.1). Wilcoxon rank-sum test was employed to compare the difference in expression of m^6^A regulators between control and breast tumor samples. The comparison of gene expression among the four subtypes was implemented by Kruskal-Wallis analysis. Univariate cox proportional hazard regression analysis was performed to evaluate the correlation between gene expression level, CNV, DNA methylation level and survival time using the *coxph* function with *survival* R package. Kaplan-Meier survival analyses and log-rank test were performed for comparison of survival time between the two clusters which was processed with *survival* R package (51).

## Results

### Alterations of m^6^A Regulators Exhibited Prognostic Values in Breast Cancer

**Figure 1:**
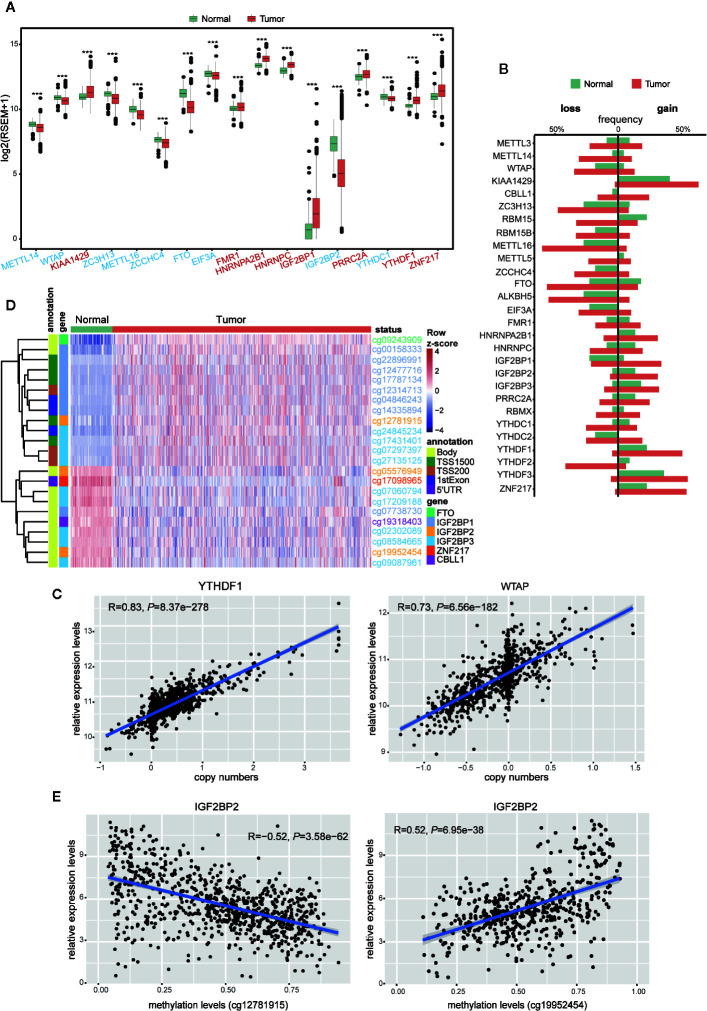
Expressions and genetic variations of m^6^A regulators in breast cancer. **(A)** Boxplot showing the m^6^A regulators with highly significant difference in their RNA expression between normal and tumor samples (***P < 0.001). Different colors of axis labels stand for the changing direction of gene expression in tumor, with red labels representing upregulation and blue labels representing downregulation. **(B)** Frequencies of the copy number gain/loss of each m^6^A regulator in normal and tumor samples. **(C)** Correlation analysis between the gene expression levels and copy numbers of two representative genes (YTHDF1 and WTAP) with the highest correlation coefficients. **(D)** Unsupervised hierarchical clustering heatmap showing the beta-values of 23 differentially methylated DNA probes between normal and tumor samples. **(E)** Correlation analysis between IGF2BP2 expression level and its DNA methylation levels at two sites (cg12781915 and cg19952454).

Accumulating evidence has confirmed that aberrant expression of m^6^A regulators is associated with tumorigenesis and progression in multiple cancers. Thereby, we asked whether this phenomenon could be observed in breast cancer. At present, 28 genes have been identified as m^6^A regulators due to their direct or indirect functions in m^6^A deposition, removal, or recognition (**Figure S1A**). First, we examined the expression of those m^6^A regulators in breast cancer and normal samples. Among them, 17 out of 28 genes exhibited significantly differential expression (*P* < 0.001), including KIAA1429, FMR1, HNRNPA2B21, HNRNPC, IGF2BP1, PRRC2A, YTHDF1, ZNF217 being upregulated and METTL14, WTAP, ZC3H13, METTL16, ZCCHC4, FTO, EIF3A, IGF2BP2, YTHDC1 being downregulated in breast cancer samples (**Figure 1A** and **Figure S1B**), suggesting their potential involvement in tumorigenesis of breast cancer.

To our knowledge, gene expression could be manipulated by multi-layered genomic features, such as DNA mutation, CNV, DNA methylation, and miRNA expression. To find out the abnormal regulatory elements for each m^6^A regulator in breast cancer, comparisons were implemented sequentially in breast cancer *versus* normal samples. The frequencies of gene mutation in all 28 m^6^A gene regulators were relatively low (**Table S2**). In contrast, their CNVs were prevalent for most m^6^A regulators. Particularly, in contrast to their low CNV frequencies (< 5%) in normal samples, CBLL1, METTL14, RBM15, IGF2BP1, YTHDC1, and YTHDF2 exhibited more than 20% difference in their frequencies of CNVs between tumor and normal samples (**Figure 1B** and **Table S2**). Next, we performed correlation analysis between copy numbers and gene expression levels to evaluate the possible effect of CNVs on gene expression. Eight regulators, including KIAA1429, METTL16, WTAP, ZCCHC4, ALKBH5, YTHDF1, YTHDF2, and YTHDF3 exhibited significant correlations (R > 0.6) between gene expression levels and copy numbers in breast tumors (**Figure 1C** and **Table S3**). It indicated that CNVs of these eight genes might be one of the causal factors to perturb their gene expression in the tumors.

In parallel, we also compared the DNA methylation levels of m^6^A regulators between tumor and normal samples. Among the 593 probes located in these 28 genes, 23 probes located on 6 genes, including CBLL1, FTO, IGF2BP1, IGF2BP2, IGF2BP3, and ZNF217, showed significant differences in their methylation patterns between tumor and normal samples (|Δbeta-value| > 0.2 and *P* < 0.05) (**Figure 1D**). Correlation analysis between the levels of DNA methylation and gene expression was further performed with all the breast cancer samples. Significant correlation (|R| > 0.3 and *P* < 0.05) was observed in WTAP, ZC3H13, ZCCHC4, FTO, ALKBH5, YTHDC2, IGF2BP2, and IGF2BP3, implying a possible role of DNA methylation in shaping their gene expression in breast cancer (**Table S4**). Markedly, negative correlation with gene expression levels was solely observed in the probes located on potential promoter regions, while positive correlation existed in gene body and 3’UTR regions only (**Figure 1E** and **Table S4**). This phenomenon implied that the DNA methylation in different genomic regions might have opposite effects on the gene expression (52, 53). In terms of miRNA regulation, despite the positive and negative relation observed between miRNA and m^6^A regulator expression, their correlation seemed to be weaker than that with copy number and DNA methylation (**Table S5**).

**Figure 2:**
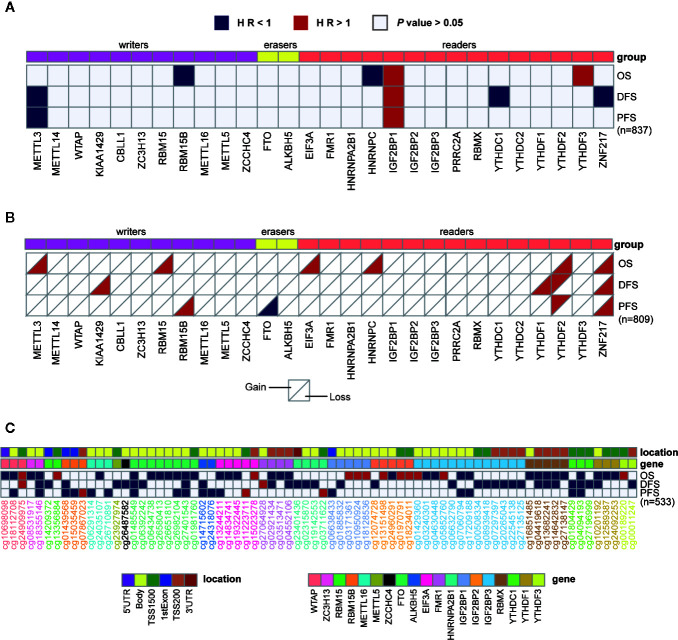
Univariate cox regression analysis of m^6^A regulators. **(A–C)** Univariate cox regression analysis of the association between overall survival (OS), disease-free survival (DFS), or progression-free survival (PFS) and gene expressions **(A)**, copy number variations **(B),** or DNA methylation levels **(C)**. Blue box, protective factors (HR < 1 and *P* < 0.05); Red box, risky factors (HR > 1 and *P* < 0.05); white box, *P* > 0.05. The sample size used in each cox regression analysis was marked in brackets.

Given the roles of m^6^A regulators in predicting prognosis observed in various cancers, we further sought to explore their potential prognostic values in breast cancer. Univariate cox regression analysis was performed with gene expression, copy number, and DNA methylation. In terms of gene expression, METTL3, RBM15B, HNRNPC, YTHDC1, and ZNF217 appeared to be protective factors with HR < 1, while IGF2BP1 and YTHDF3 were risky genes with HR > 1. Notably, higher expression of IGF2BP1 was a risky factor for overall survival (OS), disease-free survival (DFS) and progression-free survival (PFS) (**Figure 2A**). Additionally, it turned out that CNVs of m^6^A regulators also had prognostic values. Thereinto, copy number loss of FTO was a protective factor, while copy number gain of another 9 m^6^A regulators marked a worse prognosis (**Figure 2B**). Particularly, copy number loss of ZNF217 was associated with shorter OS, DFS and PFS of breast cancer patients. Regarding DNA methylation, we identified a total of 74 CpG sites located on 19 genes whose DNA methylation levels were associated with the OS, DFS or PFS of breast cancer patients (**Figure 2C**). Most of the methylation sites exhibited protective roles in prognosis. Conversely, higher methylation levels of 16 CpG sites located on WTAP, RBM15B, EIF3A, FMR1, HNRNPA2B1, IGF2BP1, IGF2BP2, IGF2BP3, and YTHDF3 were associated with poor prognosis. Intriguingly, although located on the same genes, methylation levels of distinct sites had opposite roles in predicting prognosis. Inconsistently, all significantly predictive methylation sites on FTO and YTHDC1 exhibited protective values in prognosis.

**Figure 3:**
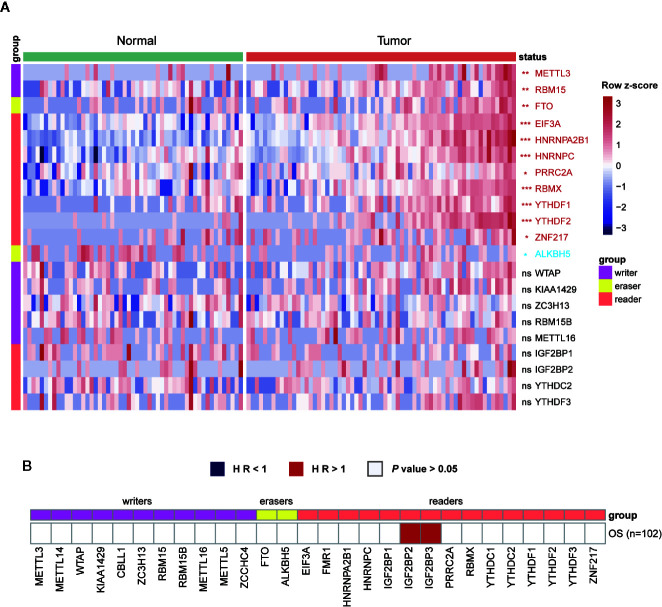
Expressions and survival analyses of m^6^A regulators at protein level. **(A)** Comparison of m^6^A regulators between tumor and normal samples at protein level. The change direction was exhibited by label colors, with red representing for upregulation and blue representing for downregulation. ns, *P* > 0.05; *P < 0.05; **P < 0.01; ***P < 0.001. **(B)** Univariate cox regression analysis of the association between protein expression levels and OS of patients. Blue box, protective factors (HR < 1 and *P* < 0.05); Red box, risky factors (HR > 1 and *P* < 0.05); white box, *P* > 0.05.

In addition to the genetic and transcriptional alterations of m^6^A regulators, we further explored whether we could detect any changes at protein level. By comparing tumor and normal samples, we found that six proteins were differentially expressed in breast cancer significantly (*P* < 0.001), including EIF3A, HNRNPA2B1, HNRNPC, RBMX, YTHDF1, YTHDF2 (**Figure 3A**). Among them, three genes (HNRNPA2B1, HNRNPC, YTHDF1) exhibited consistently aberrant expression in both RNA and protein levels while EIF3A had reverse change direction in RNA and protein levels. Besides, we performed cox regression analysis to evaluate the prognostic values of m^6^A regulators at protein level (**Figure 3B**). As a result, IGF2BP2 and IGF2BP3 showed significant prognostic values of being risk factors (HR > 1) for OS of breast cancer patients.

Together, we offered a comprehensive view of genetic, transcriptional, and post-transcriptional alterations of 28 known m^6^A regulators in breast cancer, indicative of their possible roles in tumorigenesis and diverse regulatory mechanisms. Moreover, a few genes were identified to be potential predictors for patient survival based on their changes in gene expression, copy numbers, DNA methylation, or protein levels.

### Diverse Expression Patterns and Regulations of m^6^A Regulators Among the Four Subtypes Revealed the Unique Characters of Basal-Like Subtype

Given the undeniable diversity in molecular mechanisms and clinical characteristics among the four subtypes of breast cancer, we next explored whether those m^6^A regulators exhibited any differences among them. First, by comparing the protein expression of all regulators, only IGF2BP2 exhibited differential expression among the four subtypes (**Figure S2**).

**Figure 4:**
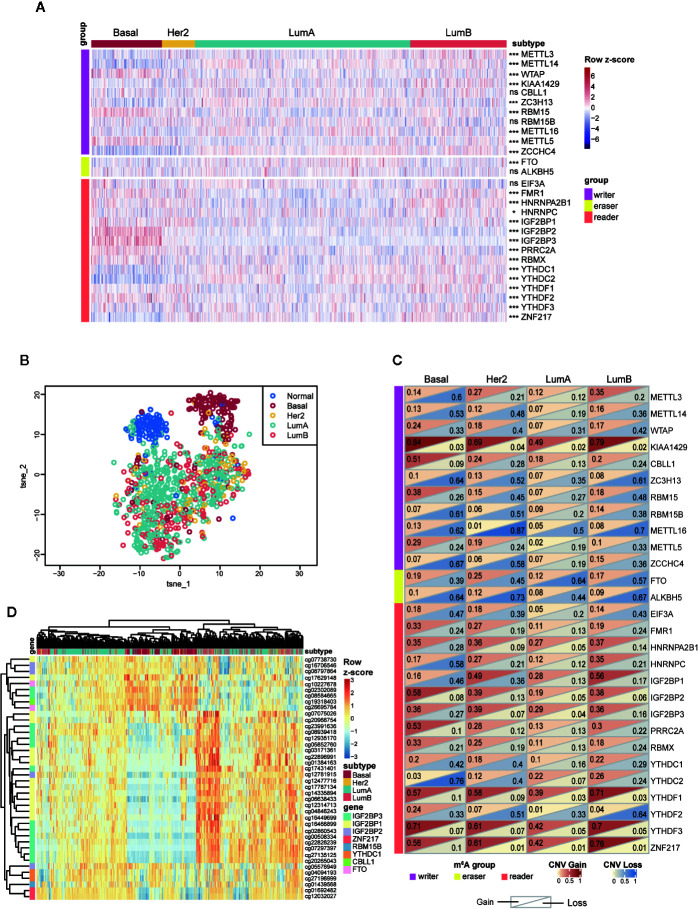
Expressions and genetic variations of m^6^A regulators among the four subtypes in breast cancer. **(A)** Heatmap showing the expression of m^6^A regulators and their differences among the four subtypes. ns, *P* > 0.05; *P < 0.05; **P < 0.01; ***P < 0.001. **(B)** t-SNE plot of normal and breast tumor samples showing the separation of normal and Basal-like samples from other groups. The colors were assigned according to sample type. **(C)** Frequencies of copy number gain/loss of m^6^A regulators in each subtype. The upper triangle of a single rectangle displays the frequency of copy number gain of each gene in each subtype, and the lower triangle displays the frequency of copy number loss. **(D)** Unsupervised hierarchical clustering analysis for four subtypes of tumors based on 40 highly variable DNA methylation probes (SD > 0.2). LumA, luminal A; LumB, luminal B; Her2, HER2-enriched; Basal, Basal-like.

To further investigate the variance among different subtypes, we turned to compare them at the transcriptional level. Ultimately, we found that 23 out of 28 m^6^A regulators exhibited significantly distinct expression levels among the four subtypes (**Figure 4A**). Next, to further explore and visualize the dispersion of m^6^A regulators in all subtypes, we adopted t-SNE method to reduce the high dimensional expression data into a lower-dimensional subspace. This result showed that breast cancer samples could be segregated from normal samples judged by the expression of m^6^A regulators. Strikingly, basal-like subtype displayed evident segregation from another three subtypes as well (**Figure 4B**). These results implied that the function of those m^6^A regulators in the four subtypes varied from each other. Particularly, basal-like subtype might exploit unique regulatory mechanisms in malignant progression driven by RNA m^6^A modification.

Next, we examined the frequencies of copy number gain/loss in each subtype, respectively (**Table S6**). Although most genes showed high frequencies of CNV without apparent discrepancy among the four subtypes, we noticed that several genes involving CBLL1, RBM15, PRRC2A exhibited particularly high frequencies of copy number gain event in basal-like subtype. Of note, 51.1% of basal-like samples contained copy number gain of CBLL1, and other subtypes showed much lower proportion (23.9% of HER2, 18.3% of luminal A, and 19.8% of luminal B). Additionally, the frequency of copy number loss in METTL3 was higher (60.0%) in basal-like subtype than that in another three subtypes (HER2: 20.9%; luminal A: 11.6%; luminal B: 20.3%); copy number loss of YTHDC2 occurred in 75.6% of basal-like tumors while significantly less in other samples (HER2: 40.3%; luminal A: 7.2%; luminal B: 24.0%) (**Figure 4C**). In short, CNVs of m^6^A regulators were prevalent in breast tumors, and the basal-like subtype exhibited the highest frequencies compared to other subtypes.

Next, DNA methylation status of the m^6^A regulators was compared among the four subtypes. Among all the probes detected in the 28 regulators, we observed that most of the CpG loci exhibited similar methylation levels among all samples (**Figure S3**). Therefore, only the highly variable methylation loci that met the criteria of standard deviation greater than 0.2 (SD > 0.2) across all tumor samples were included for subsequent analysis. Unsupervised hierarchical clustering was performed on both samples and probes to reveal the diverse methylation patterns among tumor samples. As shown in **Figure 4D**, DNA methylation patterns of a cluster of CpG sites on IGF2BP1, IGF2BP2, and IGF2BP3 exhibited prominent variance among all clustered groups. As for the clustering results, the sharpest distinction was drawn between basal-like and other subtypes. Nevertheless, parts of luminal samples were clustered together with most basal-like samples and they shared similarities in methylation levels. The discrete distribution of luminal samples reflected the noticeable intragroup heterogeneity within luminal A/B subtype revealed by the DNA methylation levels of m^6^A regulators.

In general, the above results indicated that most m^6^A regulators examined in this study possessed distinct molecular characteristics among the four subtypes. Particularly, the basal-like subtype displayed a unique feature in the aspect of gene expression, CNV and DNA methylation. Of note, DNA methylation analysis distinguished a cluster of samples consisting of basal-like subtype and a part of luminal samples due to their similar DNA methylation patterns.

### Subclassification of Luminal Subtype Breast Cancers Based on DNA Methylation of m^6^A Regulators

**Figure 5:**
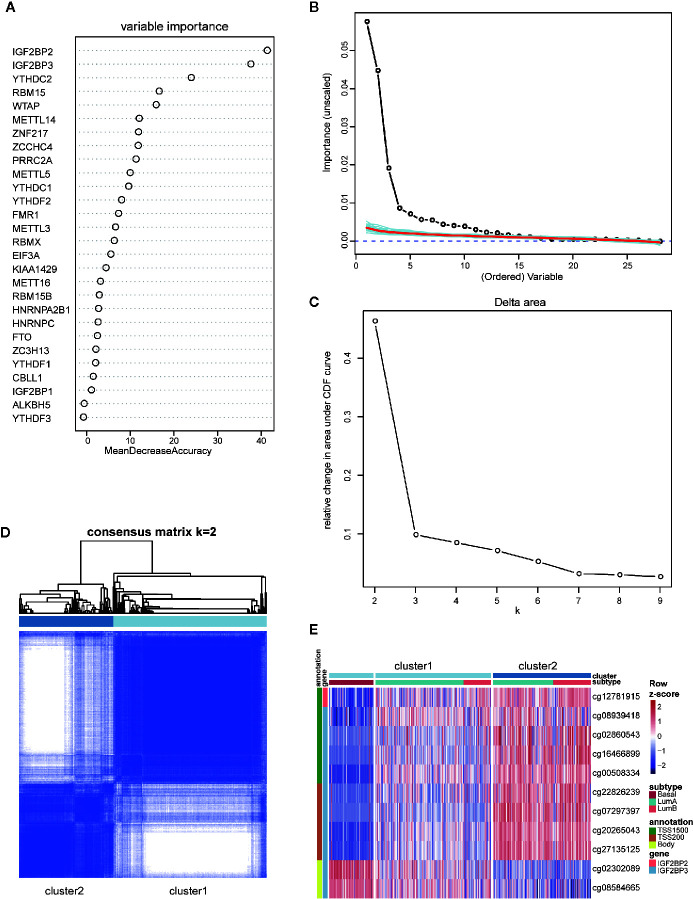
Identification and consensus clustering analysis of basal-featured m^6^A regulators. **(A)** Random forest analysis ranking the importance of m^6^A regulators in basal subtype segregation based on their gene expression levels. **(B)** Importance spectrum plots for optimizing the number of relevant variables according to random forest analysis. **(C)** Relative change in area under cumulative distribution function (CDF) curve based on results of consensus clustering for k = 2 to 9. **(D)** Consensus clustering matrix for k = 2. **(E)** Heatmap showing the methylation levels of 11 probes utilized for samples classification in the two clusters.

Although it is widely accepted that basal-like breast cancers have the highest tendency of recurrence and metastasis, some patients allocated to luminal subtype also suffer from early recurrence and metastasis which poses a big challenge in clinical practice. Given the unique profile of m^6^A regulators in basal-like subtype and high intragroup heterogeneity within luminal subtype, we asked if the m^6^A features of basal-like subtype could be applied to subclassify the luminal subtype and to distinguish the luminal samples which resembled basal-like subtype in the recurrent and metastatic property. To address this question, we firstly made efforts to obtain the basal-like subtype-featured m^6^A regulators. Based on the gene expression of 28 regulators, random forest machine learning was used to rank the gene importance and varSelRF method for variable selection. We consequently identified four genes (IGF2BP2, IGF2BP3, YTHDC2, and RBM15) as important predictors in distinguishing basal-like subtype from other subtypes (**Figures 5A, B**). Then, the expression values of those four genes were imported to consensus clustering analysis for both basal-like and luminal breast cancers. Unexpectedly, we failed to subclassify luminal samples in this way (**Figures S4A, B**).

Our previous clustering analysis of DNA methylation sites revealed that a certain number of luminal samples exhibited similar patterns to basal-like subtype in their DNA methylation patterns of highly variable CpG loci (**Figure 4D**). Therefore, we examined the possibility of using the DNA methylation status on those four genes for patients’ subclassification. Following the criteria of large standard deviation (SD > 0.2) and high correlation with gene expression (|R| > 0.3), 11 probes located on IGF2BP2 and IGF2BP3 were screened out. Consensus clustering was subsequently implemented in R with the beta values of 11 probes, and k = 2 was the optimal result, with clustering stability increasing from k = 2 to 9 (**Figures 5C, D**). Strikingly, according to the clustering results, luminal subtypes were successfully divided into two clusters, in which cluster1 was composed of all basal-like samples and parts of luminal samples, while cluster2 was composed of luminal samples only (**Figure 5E**). As for these two clusters, the methylation patterns of 11 probes in luminal-cluster1 samples were similar to basal-like samples rather than luminal-cluster2 samples. Interestingly, both luminal A and luminal B subtypes were divided into two groups and distributed in the two clusters.

**Figure 6:**
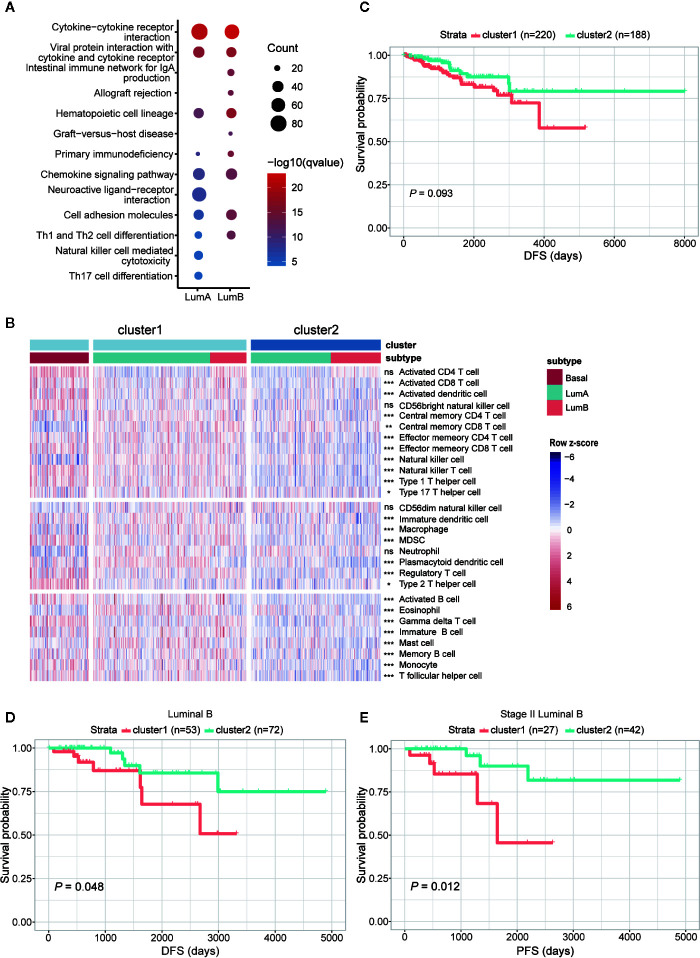
Comparison of functional and clinical relevance between the two clusters in luminal samples. **(A)** KEGG enrichment analysis of genes upregulated in cluster1 within luminal A and luminal B subtypes, respectively. **(B)** Heatmap showing relative quantities of infiltrated lymphocytes in the two clusters. **(C)** Comparison of DFS between the two clusters. **(D)** Comparison of DFS between cluster1 and cluster2 samples in luminal B subtype. **(E)** Comparison of PFS between the two clusters among patients diagnosed as stage II in luminal B subtype. The sample size of each group was marked in brackets.

To better understand the differences between the two clusters, we performed differential expression analysis and identified 2,071 upregulated and 655 downregulated genes (adjusted *P* < 0.05 and FoldChange > 1.5 or < 0.66) (**Table S7**). Next, KEGG functional enrichment analysis revealed that the upregulated genes in cluster1 of both luminal A and luminal B subtypes were mostly enriched in immune-related and cell adhesion-related pathways (**Figure 6A** and **Table S8**). To further decipher the distinct immune traits between the two clusters, we took advantage of GSVA method to evaluate the relative quantity of immune cell populations infiltrated in each sample. The results showed that samples in cluster1, similar to that in basal-like subtype, had higher enrichment in most kinds of immune cells than those in cluster2 (**Figure 6B**).

Given that cluster1 possessed similar m^6^A features with basal-like subtype and higher expression of immune-related genes, we further examined their clinical relevance in the two clusters. Comparison of survival status revealed that patients fitting in cluster1 had worse DFS than those in cluster2 (**Figure 6C**), but the difference of OS and PFS between the two clusters was indistinct (**Figures S5A, B**). To rule out the impact of different subtypes on prognosis, we next compared the two clusters within each subtype separately. It turned out that within luminal B subtype, patients in cluster1 had worse DFS than cluster 2 (**Figure 6D**), although no significant difference was observed in luminal A subtype (**Figures S5C–E**). Furthermore, in further consideration of the impact of disease stage to patient prognosis, comparisons were processed within each stage of luminal B subtype. Consequently, the most significant difference in both DFS and PFS was observed within the patients diagnosed with stage II of luminal B subtype (**Figures 6E**, **S5F–G**).

Overall, we identified basal-like subtype-featured m^6^A regulators, and further utilized their methylation patterns to successfully subclassify the luminal A/B tumors into two clusters, respectively. In line with the enrichment of immune-related genes, cell adhesion molecules and higher enrichment of tumor infiltrating lymphocytes, cluster1 samples, especially those allocated to luminal B subtype, had higher risk of disease recurrence.

## Discussion

With our increasing knowledge of m^6^A methylation in modulating RNA metabolism, how dysregulated m^6^A is involved in cancer has attracted much more attention than ever. Here, we examined the distinctive expression of m^6^A regulators and the multilayered regulation on them in breast cancer. Comparison among the four subtypes revealed a unique m^6^A feature of basal-like subtype from others. Furthermore, according to the DNA methylation status of 11 probes located on basal-featured m^6^A regulators, luminal subtypes were subclassified into two clusters with significantly different prognosis.

Till now, a few of studies have shown aberrant expression of five m^6^A regulators in breast cancer and clarified the molecular mechanism of ALKBH5- and ZNF217-mediated tumor occurrence (23, 25, 26, 28–30). Here we found a total of 17 m^6^A regulators exhibiting aberrant gene expression in breast cancer. Despite that some of them have been confirmed an oncogenic or tumor-suppressive role in hepatocellular carcinoma, leukemia, glioblastoma, and others (21), how these regulators are involved in the onset and progression of breast cancer remains elusive yet. Besides, we reported for the first time that CNV and DNA methylation change of m^6^A regulators might participate in tumorigenesis of breast cancer by shaping the gene expression. For instance, the highest correlation between copy numbers and gene expression levels was observed in YTHDF1, while IGF2BP2 has the strongest negative correlation between its DNA methylation and gene expression levels. Therefore, characterization of their functions and associated regulatory mechanism will shed new light into the mechanistic study of breast cancer from the viewpoint of RNA m^6^A methylation. In addition, we found that gene expression, copy numbers, DNA methylation levels and protein expression of several m^6^A regulators had significant correlation with either poor or improved disease outcomes, which could provide additional prognostic information and assist with precise medicine of breast cancer. It’s worth noting that, since gene expression and copy numbers or DNA methylation levels are not always correlated, they may have different or even opposite prognostic values. This inconsistency may arise from the complicated regulatory network of gene expression. For instance, multiple copies of a gene could be regulated differentially due to their corresponding chromatin environments (54). Furthermore, gene duplications that do not include distal regulatory elements important for the gene expression will not contribute to higher expression (55). On the other side, the effects of DNA methylation on gene expression are dependent to a large extent on the genomic locations of DNA methylation sites. Concretely, methylation in promoter region usually negatively correlates with gene expression, while methylation in the gene body does not block and might even stimulate transcription elongation (56). For example, among the 15 DNA methylation probes of IGF2BP3 identified in our study, the levels of 10 methylation occurring in the promoter region showed a strong negative correlation with gene expression, while the opposite trend was observed with the four methylation sites in the gene body. Hence, although luminal subtype could be subclassified into two clusters based on the methylation levels at specified CpG loci of IGF2P2 and IGF2BP3, we did not observe apparent difference in their gene expression levels between the two clusters.

Among the 28 regulators, IGF2BP2, IGF2BP3, YTHDC2, and RBM15 were identified as basal-like subtype-featured m^6^A regulators. Among them, IGF2BP2, IGF2BP3 and RBM15 were highly expressed in basal-like tumors while YTHDC2 was lowly expressed in them (**Figure 4A**). Consistent with our findings, Barghash et al. reported that increased expression of IGF2BP2 was regarded as a feature of basal-like subtype and correlated with short survival (57). In addition, suppressed IGF2BP2 could hinder cell proliferation and invasion in breast cancer (58). As to IGF2BP3, despite the comparable expression between breast cancer and normal samples in our data, its expression in basal-like subtype was significantly higher than that in other subtypes (**Figure 4A**). In agreement with that, tumors with higher IGF2BP3 expression were characterized by increased tumor size, advanced tumor stage, and lymph node metastasis (59). Similarly, higher protein levels of IGF2BP2 and IGF2BP3 were also proved to be associated with high-risk prognosis in our results (**Figure 3B**). Different from IGF2BP2 and IGF2BP3, although the oncogenic roles of YTHDC2 and RBM15 have been identified in colon cancer (60) and acute megakaryoblastic leukemia (61–63), their functions in breast cancer await to be identified.

Breast cancer is a complex disease with large degree of intertumoral and intratumoral heterogeneity. In recent years, molecular subtyping distinguished by gene expression profiling in breast cancer has contributed a lot to prolong patients’ survival due to the improvement in precise diagnosis and targeting therapy (6). Nevertheless, within each subtype, there still exists substantial heterogeneity and therefore requires more extensive and thorough investigation of breast cancer. In this study, in addition to the significant difference between normal and cancer samples, the performances of these m^6^A regulators in the four subtypes were distinct from each other as well. Particularly, basal-like subtype is unique in its gene expression, copy number, and DNA methylation. Given the fact that basal-like subtype is more aggressive and has a worse prognosis than other subtypes (1, 4, 5), the unique features of m^6^A regulators in basal-like subtype suggest their possible involvement in tumor invasion and metastasis. In line with that, higher expression of cell adhesion molecules was detected in samples assigned to cluster1, further indicating the correlation between m^6^A regulators and breast cancer malignancy.

Luminal breast tumors are the most common subtypes (64); meanwhile, they are also highly heterogeneous in the aspect of histology, gene expression profiles, genetic alterations, and clinical outcomes (65). Despite endocrine therapy and chemotherapy available for them, some patients of this subtype still suffer from relapse and poor prognosis (7, 66), thereby highlighting the emergent need for early prediction for those latent patients. Effective biomarkers can accurately instruct patients to access suitable therapies, thus helping advanced patients to achieve positive clinical response to treatment in a short time. As DNA is more stable than RNAs or proteins and easily quantified, DNA methylation is considered as a robust biomarker and promising biomarker for early detection and diagnosis (67). In this study, based on DNA methylation of m^6^A regulators, luminal samples were subclassified into two clusters with distinct expression levels of immune-related genes. According to previous studies, immune environment of breast tumors has profound effects on patients’ prognosis and varies among the four subtypes. Accordingly, basal-like subtype has the highest rate of TILs than other subtypes (68). In the luminal-HER2^-^ patients, a higher TIL number was associated with shorter overall survival as well (69). This is consistent with our results that cluster1 in luminal samples had higher number of TILs and worse prognosis. Since the presence of TILs indicates better sensitivity to neoadjuvant chemotherapy (69), our subclassification strategy may provide a clue to recognize those luminal tumors more suitable for neoadjuvant therapy. The unveiled immune variance within luminal subtype in our study was also illustrated in two published research. One of them performed segregation analysis of luminal group based on immune-related genes and identified three immune subtypes which owned distinct clinical characteristics (11). The other study highlighted that even within luminal A subtype, immune heterogeneity could not be ignored either, as revealed by a large-scale transcriptome analysis. Both gene expression and DNA methylation profiles were successfully applied to segregate luminal A samples into two biologically distinct subgroups with different expression patterns of immune-related genes (14). However, this method exploited a large number of partitioning genes for subclassification, which made it difficult to be translated into clinical application. By contrast, our study put forward a small gene set that could be applied to luminal subtype partition and thereby is of more practical use. Mechanistically, as sample clustering was implemented with DNA methylation of m^6^A regulators, the different enrichment of TILs in luminal tumors between the two clusters may be associated with the m^6^A RNA methylation. RNA m^6^A methylation has already been reported to be correlated with immune responses, such as T cell homeostasis (70, 71), inflammatory response (72), antiviral immunity (73–77), and anti-tumor immune response (78). Thereby, intensive mechanistic studies are necessary to uncover the mechanism of how m^6^A impacts tumor relapse or metastasis *via* its modulatory roles on immune response.

Although our strategy could be successfully applied to subclassify the luminal subtype and has predictive value in clinical, some limitations should be noted here. First, given the existing inconsistency between RNA and protein levels observed in other types of cancers (79–81), it is important to depict the performance of m^6^A regulators in breast cancers at protein level. Although the additionally obtained two proteomic datasets provided certain information about the function of m^6^A regulators in breast cancer at protein levels, the sample size was not sufficient. So the conclusions needed to be assured by well-designed experiments later on. Second, there was a lack of validation cohorts in our study. We searched out for Gene Expression Omnibus (GEO), International Cancer Genome Consortium (ICGC) and Cell-Free Epigenome Atlas (CFEA) databases to filter the datasets that provided detailed clinical information including intrinsic subtypes, survival status and supported by platform Illumina HumanMethylation450 BeadChip. While in consideration of all kinds of criteria, no proper data was available. Third, due to the limited information of miRNA-mRNA interactions available from database, miRNA-mRNA regulatory network specifically existing in breast cancer may not be completely included in our datasets. So extended interaction information, particularly breast tissue-specific data is necessary for more thorough studies. Last, the heterogeneity of breast tumors represents a formidable challenge of successful cancer treatment. Although our research has made efforts to explain the intertumoral heterogeneity in luminal subtype, the intratumoral heterogeneity remains elusive yet since the data analyzed here was obtained based on mixed cell population rather than single cells.

In summary, our study explored the alteration of m^6^A regulators at multiple levels in breast cancer and revealed their potential prognostic values. Furthermore, by taking advantage of DNA methylation of basal-featured m^6^A regulators, luminal A and luminal B subtypes were both segregated into two clusters, which are associated with different abundance of immune infiltrating lymphocytes and prognosis of patients. Together, our study expands the realm of mechanistic study in breast cancer and discovers novel strategy in subclassifying luminal subtype for the sake of personalized treatment.

## Data Availability Statement

Publicly available datasets were analyzed in this study. This data can be found here: https://xenabrowser.net/.

## Author Contributions

YN and W-MT conceived and designed the project. LY performed the bioinformatics analyses with the help of SS and CM. LY, YN, SW, FJ, and W-MT wrote the manuscript. All authors contributed to the article and approved the submitted version.

## Funding

This study was supported by the Medical Epigenetics Research Center, Chinese Academy of Medical Sciences (2019PT310017, to YN).

## Conflict of Interest

The authors declare that the research was conducted in the absence of any commercial or financial relationships that could be construed as a potential conflict of interest.

## Abbreviations

m^6^A, N^6^-methyladenosine; TCGA, The Cancer Genome Atlas; CNV, copy number variation; HER2, human epidermal growth factor 2; PR, progesterone receptor; MAF, Mutation Annotation Format; t-SNE, t-distributed Stochastic Neighbor Embedding; SD, standard deviation; CDF, cumulative distribution function; KEGG, Kyoto Encyclopedia of Genes and Genomes; TILs, tumor infiltrating lymphocytes; ssGSEA, single sample gene set enrichment analysis; TSS, Transcription Start Site; NS, not significant; OS, overall survival; DFS, disease-free survival; PFS, progression-free survival; UTR, untranslated region; HR, hazard ratio; GEO, Gene Expression Omnibus; ICGC, International Cancer Genome Consortium; CFEA, Cell-Free Epigenome Atlas.
