# Supplementary material for: RNA m6A Methylation Regulators Subclassify Luminal Subtype in Breast Cancer
Source: Front Oncol. 2021 Jan 29;10:611191. doi: 10.3389/fonc.2020.611191 (PMC7878528; doi:10.3389/fonc.2020.611191)
Supplement: Supplementary file 1 [file DataSheet_1.pdf]

Supplementary files

FIGURE S1 | Comparison of gene expressions of m^6A regulators in normal and breast cancer samples. **(A)** Pie chart showing the currently identified m^6A regulators including 11 writers, 2 erasers, and 15 readers. **(B)** Boxplot showing the m^6A regulators with less significant (*$P < 0.05$; **$P < 0.01$) or non-significant (ns, $P > 0.05$) difference in their gene expression between normal and tumor samples.

FIGURE S2 | Heatmap exhibiting the protein expression levels of all detected m^6A regulators among the four subtypes of breast cancer. Differential expression analysis was performed among four subtypes. ns, $P > 0.05$; *$P < 0.05$; **$P < 0.01$; ***$P < 0.001$.

FIGURE S3 | Hierarchal clustering heatmap showing beta values of all DNA methylation sites on m^6A regulators among the four subtypes of breast cancers.

FIGURE S4 | Consensus clustering of breast cancer samples with expression levels of 4 basal-featured m^6A regulators. **(A)** Relative change in area under cumulative distribution function (CDF) curve based on results of consensus clustering for k = 2 to 9. **(B)** Consensus clustering matrix for k = 2.

FIGURE S5 | Kaplan-Meier survival analysis between the two clusters. **(A-B)** Comparison of OS **(A)** and PFS **(B)** between the two clusters. **(C-E)** Comparison of OS **(C)**, DFS **(D)** and PFS **(E)** between the two clusters within luminal A subtype. **(F-G)** Comparison of OS **(F)** and DFS **(G)** between two clusters among patients diagnosed as stage II of luminal B subtype. The sample size of each group was marked in brackets.

Supplementary table 1. Information of datasets used in this study.

Supplementary table 2. Frequencies of copy number gain/loss and mutation of m^6A regulators in normal and tumor samples.

Supplementary table 3. Pearson correlation analysis between gene expression levels and copy numbers of m^6A regulators.

Supplementary table 4. Pearson correlation analysis between the levels of gene expression and DNA methylation of m^6A regulators filtered with criteria of |correlation coefficient| > 0.3 & P value < 0.05.

Supplementary table 5. Pearson correlation analysis between gene expression levels

of m^6A regulators and corresponding target miRNAs filtered with criteria of |correlation coefficient| > 0.2 & *P* value < 0.05.

Supplementary table 6. Frequencies of copy number gain/loss of m^6A regulators in each subtype of breast cancer.

Supplementary table 7. Differentially expressed genes between cluster1 and cluster2 samples within luminal A (sheet1) and luminal B (sheet2) subtype separately. (*see separate excel file.*)

Supplementary table 8. Top 10 most enriched KEGG pathways of the up-regulated genes in cluster1 samples with luminal A subtype (sheet1) and luminal B subtype (sheet2). (*see separate excel file.*)

A

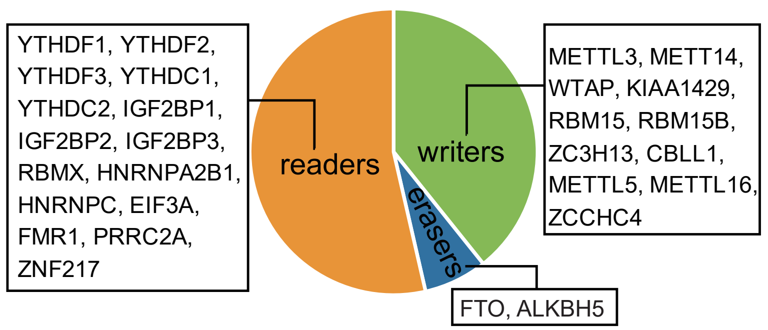
YTHDF1, YTHDF2, YTHDF3, YTHDC1, YTHDC2, IGF2BP1, IGF2BP2, IGF2BP3, RBMX, HNRNPA2B1, HNRNPC, EIF3A, FMR1, PRRC2A, ZNF217
readers
writers
erasers
METTL3, METT14, WTAP, KIAA1429, RBM15, RBM15B, ZC3H13, CBLL1, METTL5, METTL16, ZCCHC4
FTO, ALKBH5

B

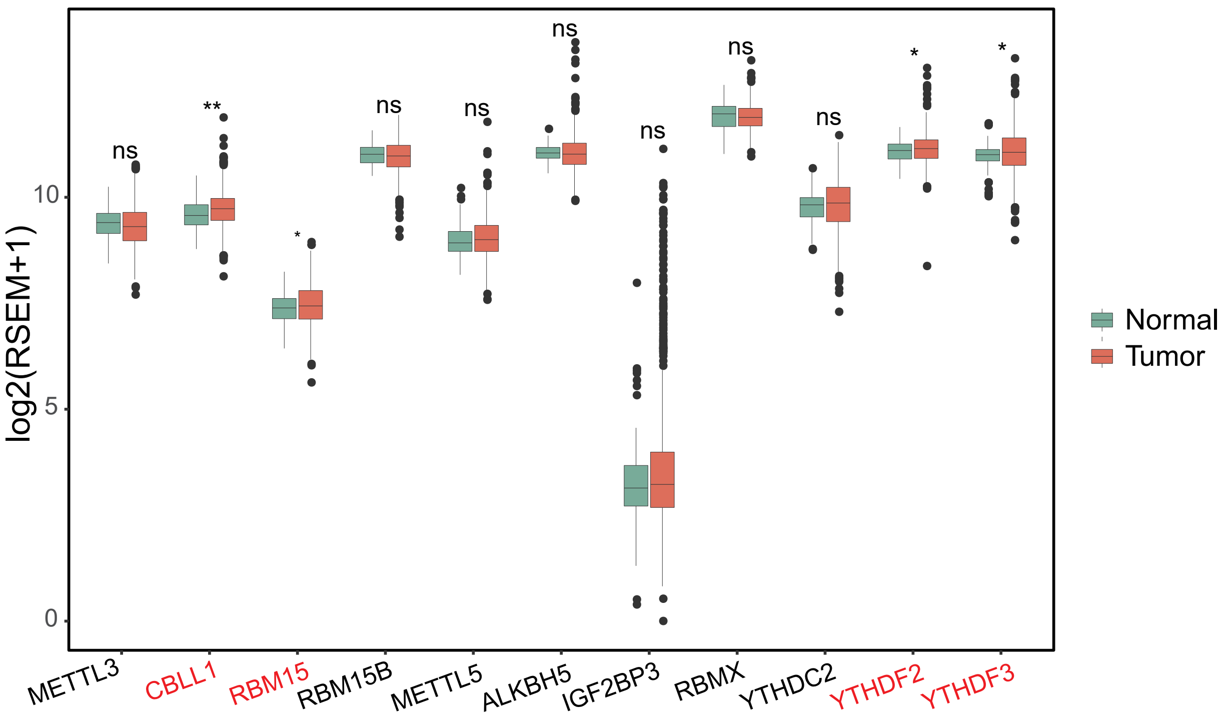
log2(RSEM+1)
0
5
10
ns
**
*
ns
ns
ns
ns
ns
ns
*
*
METTL3
CBLL1
RBM15
RBM15B
METTL5
ALKBH5
IGF2BP3
RBMX
YTHDC2
YTHDF2
YTHDF3
Normal
Tumor

A

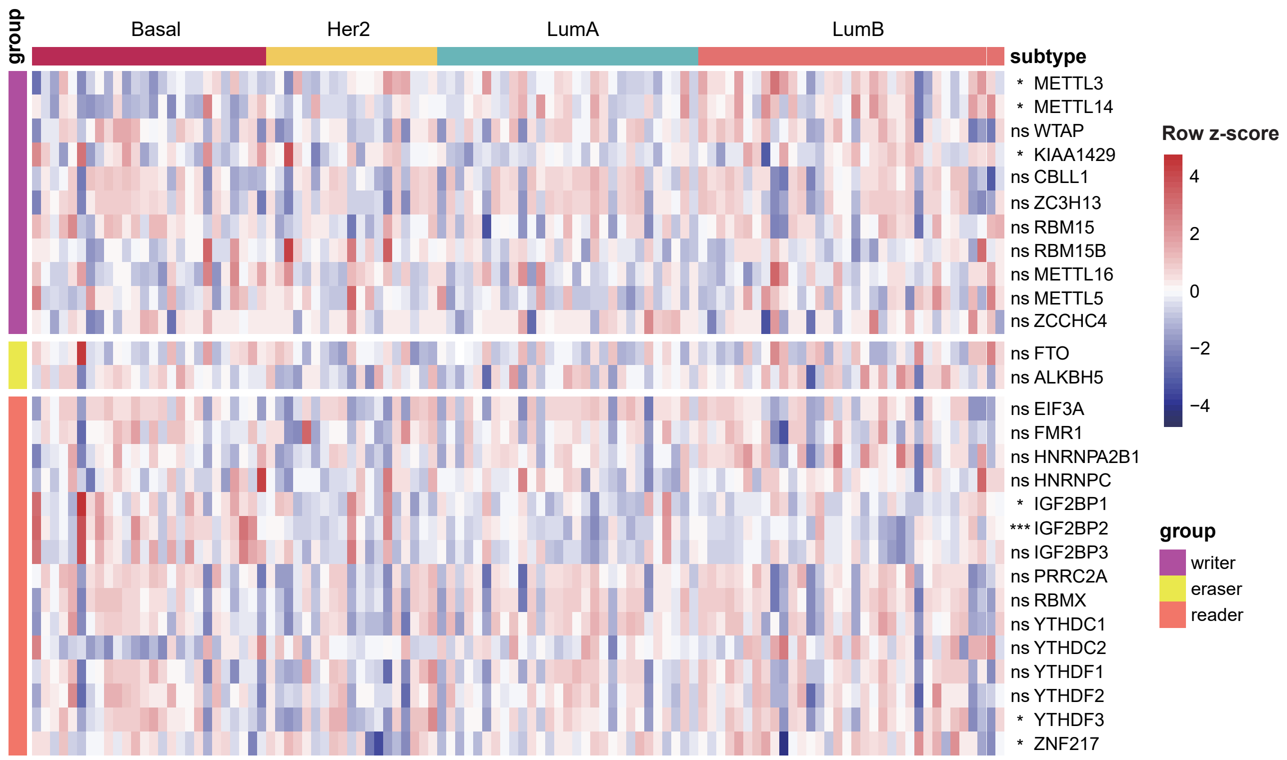

group
Basal
Her2
LumA
LumB
subtype
* METTL3
* METTL14
ns WTAP
* KIAA1429
ns CBLL1
ns ZC3H13
ns RBM15
ns RBM15B
ns METTL16
ns METTL5
ns ZCCHC4
ns FTO
ns ALKBH5
ns EIF3A
ns FMR1
ns HNRNPA2B1
ns HNRNPC
* IGF2BP1
*** IGF2BP2
ns IGF2BP3
ns PRRC2A
ns RBMX
ns YTHDC1
ns YTHDC2
ns YTHDF1
ns YTHDF2
* YTHDF3
* ZNF217
Row z-score
4
2
0
−2
−4
group
writer
eraser
reader

A

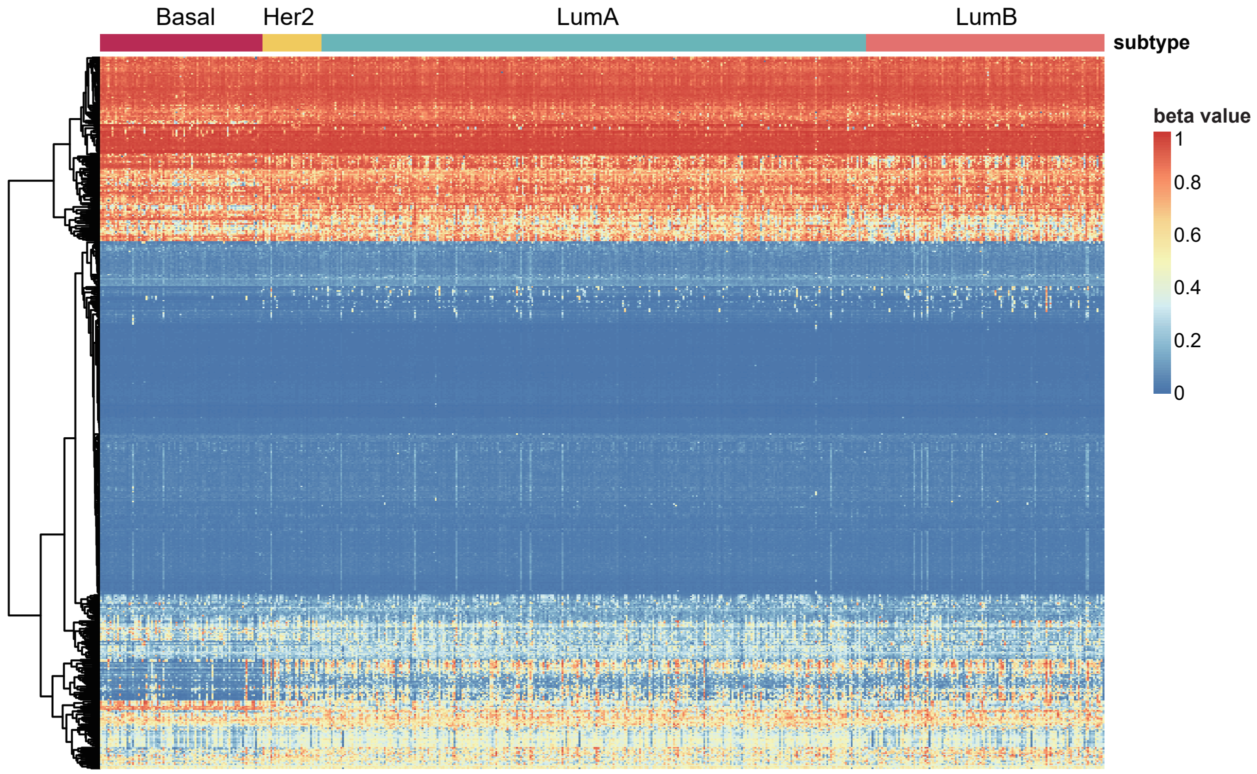

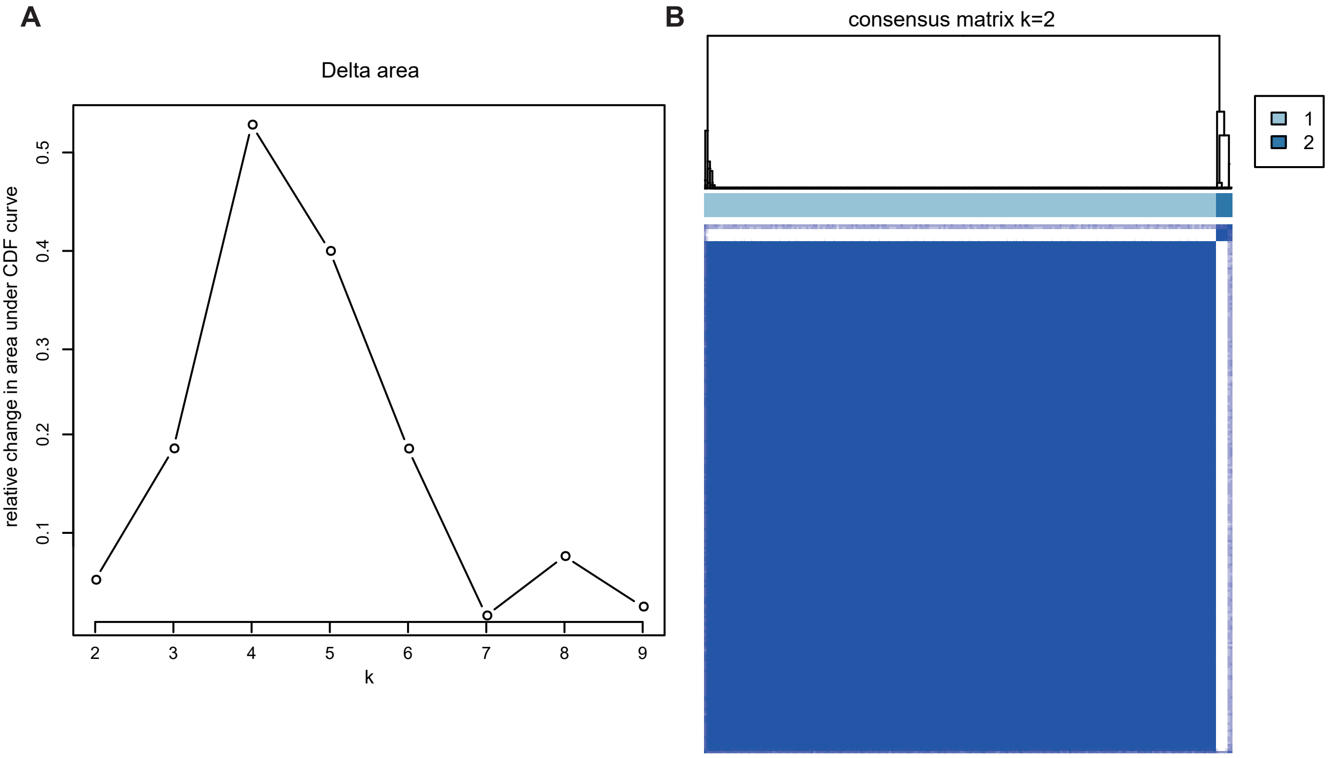
A
Delta area
relative change in area under CDF curve
0.1
0.2
0.3
0.4
0.5
2
3
4
5
6
7
8
9
k
B
consensus matrix k=2
1
2

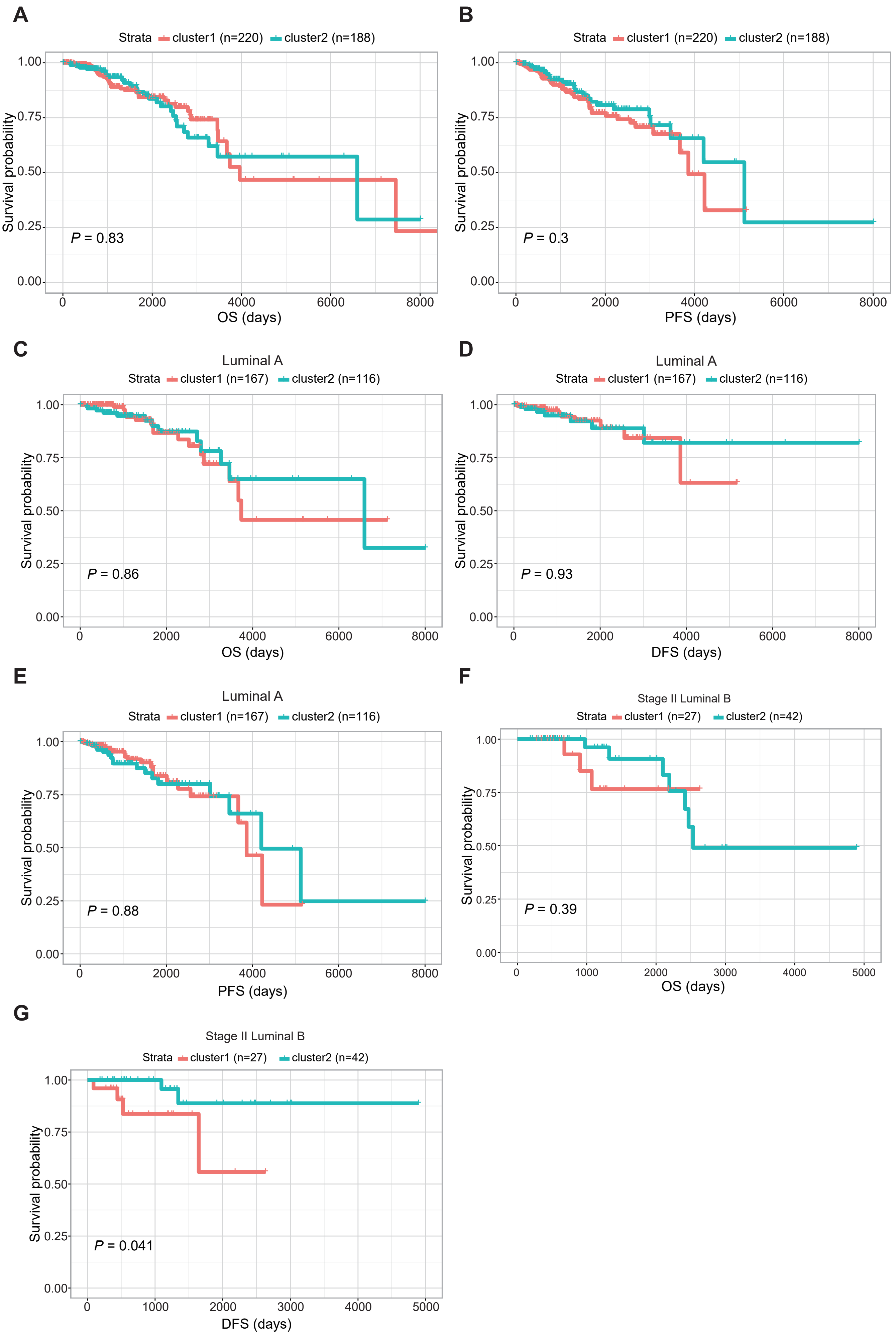
A
Strata cluster1 (n=220) cluster2 (n=188)
Survival probability
1.00
0.75
0.50
0.25
0.00
P = 0.83
0
2000
4000
6000
8000
OS (days)
B
Strata cluster1 (n=220) cluster2 (n=188)
Survival probability
P = 0.3
PFS (days)
C
Luminal A
Strata cluster1 (n=167) cluster2 (n=116)
Survival probability
P = 0.86
OS (days)
D
Luminal A
Strata cluster1 (n=167) cluster2 (n=116)
Survival probability
P = 0.93
DFS (days)
E
Luminal A
Strata cluster1 (n=167) cluster2 (n=116)
Survival probability
P = 0.88
PFS (days)
F
Stage II Luminal B
Strata cluster1 (n=27) cluster2 (n=42)
Survival probability
P = 0.39
0
1000
2000
3000
4000
5000
OS (days)
G
Stage II Luminal B
Strata cluster1 (n=27) cluster2 (n=42)
Survival probability
P = 0.041
DFS (days)

Supplementary tables

Supplementary table 1. Information of datasets used in this study.

Dataset	Normal	Basal	Her2	LumA	LumB
Gene expression (RNA-seq)	119	142	67	434	194
Phenotypes	119	142	67	434	194
DNA methylation (450k)	87	87	31	288	127
Copy number (gene-level)	22	135	67	415	192
Somatic mutation	19	106	42	301	125
miRNA expression (RNA-seq)	89	84	43	252	116
Protein level (ref. 40)	53	15	17	22	0
Protein level (ref. 41)	3	25	18	29	33

Supplementary table 2. Frequencies of copy number gain/loss and mutation of m^6A regulators in tumor and normal samples.

	gene	mutation (%)		gain (%)		loss (%)	
		normal	tumor	normal	tumor	normal	tumor
writers	METTL3	0	0.24	9.09	19.04	9.09	22.5
	METTL14	0	0.6	4.55	10.75	4.55	31.03
	WTAP	0.84	0.36	4.55	12.98	18.18	34.73
	KIAA1429	0	1.91	40.91	63.66	0	2.6
	CBLL1	0	0.12	0	24.6	4.55	16.19
	ZC3H13	0	0.72	9.09	8.41	27.27	47.71
	RBM15	0	0.24	22.73	15.45	0	33.13
	RBM15B	0	0.12	0	9.27	18.18	33.37
	METTL16	0	0.24	0	6.67	27.27	60.07
	METTL5	0	0.48	4.55	10.01	0	23.49
	ZCCHC4	0	0.36	0	8.65	18.18	34.61
erasers	FTO	0	0.48	18.18	15.7	22.73	56.37
	ALKBH5	0	0.48	0	8.9	27.27	55.01
readers	EIF3A	0	0.96	0	10.38	22.73	31.27
	FMR1	0	1.67	9.09	17.8	9.09	17.92
	HNRNPA2B1	0	1.19	13.64	31.4	0	11.37
	HNRNPC	0	0.36	13.64	19.41	9.09	22.37
	IGF2BP1	0	0.24	4.55	34.24	22.73	21.38
	IGF2BP2	0.84	1.19	13.64	31.4	4.55	6.3
	IGF2BP3	0	0.12	18.18	32.39	4.55	11
	PRRC2A	0	0.12	13.64	24.97	4.55	14.71
	RBMX	0	0.24	4.55	17.43	4.55	17.43
	YTHDC1	0.84	0.36	9.09	14.96	4.55	25.34
	YTHDC2	0.84	0.36	0	19.16	18.18	25.34
	YTHDF1	0.84	1.19	22.73	50.93	0	4.7
	YTHDF2	0	0.6	9.09	6.3	0	41.66
	YTHDF3	0	0.6	36.36	55.13	0	5.69
	ZNF217	0	1.19	22.73	54.14	0	2.35

Supplementary table 3. Pearson correlation analysis between gene expression levels and copy numbers of m^{6}A regulators.

	gene	correlation coefficient	*P* value
writers	METTL3	0.45	7.70E-55
	METTL14	0.48	3.43E-63
	WTAP	0.73	6.56E-182
	KIAA1429	0.63	1.33E-122
	CBLL1	0.42	2.09E-48
	ZC3H13	0.49	6.09E-65
	RBM15	0.34	1.03E-29
	RBM15B	0.54	3.78E-84
	METTL16	0.66	2.03E-134
	METTL5	0.4	3.71E-43
	ZCCHC4	0.63	1.35E-119
erasers	FTO	0.41	3.22E-44
	ALKBH5	0.69	1.42E-155
readers	EIF3A	0.43	4.24E-49
	FMR1	0.19	2.13E-10
	HNRNPA2B1	0.29	7.56E-23
	HNRNPC	0.32	1.01E-26
	IGF2BP1	0.22	9.63E-14
	IGF2BP2	0.19	1.46E-10
	IGF2BP3	0.06	0.059605135
	PRRC2A	0.53	3.33E-79
	RBMX	0.08	0.007108901
	YTHDC1	0.47	4.63E-59
	YTHDC2	0.57	2.48E-93
	YTHDF1	0.83	8.37E-278
	YTHDF2	0.63	2.69E-121
	YTHDF3	0.63	1.09E-118
	ZNF217	0.43	6.31E-51

Supplementary table 4. Pearson correlation analysis between the levels of gene expression and DNA methylation of m^{6}A regulators filtered with criteria of |correlation coefficient| > 0.3 & *P* value < 0.05.

	probeID	gene	correlation coefficient	*P* value	group
writers	cg10698098	WTAP;WTAP;WTAP	-0.42	2.92E-24	5'UTR;5'UTR;TSS1500
	cg11785509	WTAP;WTAP;WTAP	-0.31	5.83E-13	5'UTR;5'UTR;5'UTR
	cg13623384	WTAP	0.37	1.05E-18	3'UTR
	cg17212720	WTAP;WTAP;WTAP	-0.41	1.37E-22	TSS200;TSS200;TSS1500
	cg24665647	WTAP;WTAP;WTAP	0.31	3.04E-13	Body;Body;Body
	cg19294256	ZC3H13	-0.31	2.50E-13	TSS200
	cg13499600	ZCCHC4	-0.44	6.11E-27	3'UTR
	cg19289767	ZCCHC4	-0.36	5.81E-18	TSS200
erasers	cg10227678	FTO	-0.36	4.72E-18	Body
	cg05395061	ALKBH5	-0.37	6.41E-19	TSS200
	cg13204529	ALKBH5	-0.33	4.76E-15	TSS200
	cg14515250	ALKBH5	-0.35	1.05E-16	TSS1500
	cg22247039	ALKBH5	-0.35	4.47E-17	TSS200
reader	cg08434396	FMR1	-0.31	2.09E-13	Body
	cg12781915	IGF2BP2;IGF2BP2	-0.49	6.97E-33	TSS1500;TSS1500
	cg15487251	IGF2BP2;IGF2BP2	-0.64	2.62E-61	TSS1500;TSS1500
	cg19952454	IGF2BP2;IGF2BP2	0.52	6.95E-38	Body;Body
	cg00508334	IGF2BP3	-0.49	7.85E-33	TSS1500
	cg02302089	IGF2BP3	0.42	1.15E-24	Body
	cg02860543	IGF2BP3	-0.33	1.07E-14	TSS1500
	cg04630448	IGF2BP3	0.36	4.10E-18	Body
	cg07297397	IGF2BP3	-0.35	3.52E-17	TSS200
	cg08153160	IGF2BP3	-0.36	3.78E-18	Body
	cg08584665	IGF2BP3	0.34	1.95E-15	Body
	cg08939418	IGF2BP3	-0.46	6.80E-29	TSS1500
	cg09087961	IGF2BP3	0.56	1.15E-45	Body
	cg12601843	IGF2BP3	-0.47	1.42E-30	TSS1500
	cg16466899	IGF2BP3	-0.38	3.20E-19	TSS1500
	cg20265043	IGF2BP3	-0.43	1.26E-25	TSS200
	cg22826239	IGF2BP3	-0.38	8.16E-20	TSS200
	cg24845234	IGF2BP3	-0.47	1.92E-30	1stExon
	cg27135125	IGF2BP3	-0.43	4.68E-25	TSS200
	cg24145369	YTHDC2	0.36	1.44E-17	3'UTR
	cg08445782	ZNF217	-0.31	3.29E-13	Body

Supplementary table 5. Pearson correlation analysis between gene expression levels of m^{6}A regulators and corresponding target miRNAs filtered with criteria of |correlation coefficient| > 0.2 & *P* value < 0.05.

	mRNA	miRNA	Correlation coefficient	*P* value
writers	METTL14	hsa-miR-4443	-0.29	3.18E-08
	WTAP	hsa-miR-378a-3p	0.20	6.62E-07
	ZCCHC4	hsa-miR-455-3p	-0.25	6.03E-10
erasers	FTO	hsa-miR-30c-2-3p	0.27	4.22E-11
readers	HNRNPA2B1	hsa-miR-15a-5p	0.33	6.51E-16
	HNRNPA2B1	hsa-miR-107	0.32	2.62E-15
	HNRNPA2B1	hsa-miR-204-5p	-0.34	2.74E-15
	HNRNPA2B1	hsa-miR-15b-5p	0.33	3.12E-16
	HNRNPA2B1	hsa-miR-195-5p	-0.24	5.14E-09
	HNRNPA2B1	hsa-miR-140-3p	-0.27	4.65E-11
	HNRNPA2B1	hsa-miR-4724-5p	0.25	3.71E-07
	HNRNPC	hsa-miR-495-3p	-0.29	2.60E-12
	HNRNPC	hsa-miR-139-3p	-0.30	2.68E-13
	HNRNPC	hsa-miR-7-1-3p	0.23	2.15E-08
	HNRNPC	hsa-miR-3199	-0.24	2.43E-08
	IGF2BP2	hsa-miR-18a-5p	0.31	2.16E-14
	IGF2BP2	hsa-miR-129-5p	0.30	1.92E-12
	IGF2BP3	hsa-miR-98-5p	0.25	1.18E-09
	IGF2BP3	hsa-miR-129-5p	0.21	7.16E-07
	IGF2BP3	hsa-let-7g-5p	0.22	1.47E-07
	IGF2BP3	hsa-let-7i-5p	0.28	7.52E-12
	IGF2BP3	hsa-miR-142-5p	0.36	2.26E-19
	IGF2BP3	hsa-miR-9-5p	0.30	1.35E-13
	YTHDC1	hsa-miR-30a-3p	0.23	2.62E-08
	YTHDC1	hsa-miR-195-5p	0.23	2.35E-08
	YTHDC1	hsa-miR-497-5p	0.21	4.16E-07
	YTHDC1	hsa-miR-22-5p	-0.26	1.21E-10
	YTHDC1	hsa-miR-7156-5p	-0.23	8.55E-06
	YTHDF1	hsa-miR-139-5p	-0.24	4.52E-09
	ZNF217	hsa-miR-378a-5p	-0.26	2.08E-10

Supplementary table 6. Frequencies of copy number gain/loss of m^{6}A regulators in each subtype of breast cancer.

	gene	Gain (%)				Loss (%)			
		Basal	Her2	LumA	LumB	Basal	Her2	LumA	LumB
writers	METTL3	14.07	26.87	11.81	35.42	60.00	20.90	11.57	20.31
	METTL14	13.33	11.94	7.23	16.15	53.33	47.76	18.55	36.46
	WTAP	24.44	17.91	6.75	16.67	32.59	40.30	31.08	42.19
	KIAA1429	84.44	68.66	48.92	79.17	2.96	4.48	2.41	2.08
	CBLL1	51.11	23.88	18.31	19.79	8.89	28.36	13.01	23.96
	ZC3H13	10.37	13.43	7.23	7.81	63.70	52.24	35.42	61.46
	RBM15	37.78	14.93	7.23	17.71	25.93	44.78	26.75	47.92
	RBM15B	6.67	5.97	8.67	13.54	60.74	50.75	19.52	38.02
	METTL16	12.59	1.49	4.82	8.33	62.22	86.57	50.36	70.31
	METTL5	28.89	19.40	2.41	9.90	23.70	23.88	18.80	33.33
	ZCCHC4	6.67	5.97	6.99	14.58	67.41	58.21	19.28	36.46
erasers	FTO	19.26	25.37	12.29	17.19	39.26	44.78	63.61	56.77
	ALKBH5	9.63	11.94	7.95	9.38	64.44	73.13	43.61	66.67
writers	EIF3A	17.78	17.91	5.06	14.06	47.41	38.81	19.52	42.71
	FMR1	33.33	26.87	10.84	18.75	23.70	19.40	12.77	24.48
	HNRNPA2B1	34.81	35.82	26.99	36.98	28.15	8.96	5.06	14.06
	HNRNPC	17.04	26.87	11.81	34.90	57.78	20.90	11.81	20.83
	IGF2BP1	15.56	49.25	27.71	56.25	45.93	35.82	13.25	16.67
	IGF2BP2	57.78	38.81	18.55	38.02	8.15	13.43	4.58	6.25
	IGF2BP3	35.56	38.81	28.67	35.94	26.67	7.46	4.10	16.15
	PRRC2A	52.59	28.36	13.01	30.21	10.37	11.94	13.25	21.88
	RBMX	33.33	25.37	10.84	17.71	21.48	19.40	12.77	23.96
	YTHDC1	20.00	17.91	9.64	21.88	42.22	40.30	15.66	29.17
	YTHDC2	2.96	11.94	22.41	26.04	75.56	40.30	7.23	23.96
	YTHDF1	57.04	58.21	38.55	70.83	9.63	8.96	3.13	3.13
	YTHDF2	23.70	7.46	1.45	4.17	33.33	50.75	32.53	64.06
	YTHDF3	71.11	61.19	41.93	70.31	7.41	7.46	5.06	5.21
	ZNF217	55.56	61.19	42.41	76.04	10.37	1.49	0.72	0.52
